# Nursing Progress and Its Developments

**Published:** 1918-05-11

**Authors:** 


### THE MATRONS' AND SISTERS' DEPARTMENT.

### XIII.—NURSING PROGRESS AND ITS DEVELOPMENTS.

### The Local Centre System for London.

The continued activity of the College of Nursing in the provinces has brought to light the desire of trained nurses for a professional stimulus and their ability to organise themselves for professional and social purposes. The College, great in its aims and already powerful in its central organisation, is leading to a form of self-expression among nurses which they are fully prepared to use. However splendid may be the central achievements of the College, these can hardly outweigh the further boon it is beginning to confer in setting nurses to organise schemes for their own benefit in various localities. It is bringing a wholesome current of interest and emulation into a profession too much cut off by the circumstances of work from the outside world.

Why should the benefits of local centres be confined to nurses who live outside the metropolis? In the vast aggregation of boroughs which makes up the greatest city in the world there are gathered probably, in normal times, more nurses than in the rest of the Empire. If it be good, as is now evident, for nurses in provincial centres to have a local corporate existence under the ægis of the College, to elect their own officers, think out plans for the benefit of their members, establish a clubhouse, organise post-graduate lectures, and hold meetings for discussion and recreation, would not these things be good also for nurses dwelling in various far-sundered parts of London? It is true that Londoners have opportunities which are denied to more remote members of the College, inasmuch as they can all perhaps reach the headquarters of the College in an hour or two. But it must always be impossible to fix upon a district which shall be central for all London. Hampstead, Richmond, Marylebone, Bethnal Green, how impracticable to study the convenience of all such centres simultaneously! An occasional visit to headquarters, such as perhaps members from the provinces can manage with less fatigue than dwellers in outlying parts of London, is a poor substitute for a club-room in the next street or within ten minutes by Tube or omnibus. We believe that there is ample material in a dozen quarters in and round London for the formation of centres of the College which in influence and practical use might rival those formed in large towns at a distance.

The more perfect and beneficial the central organisation of the College becomes, the more needful it will be to draw its members into the path of self-government. Both in institutions and in private work nurses are too much debarred by the conditions under which they work from the free play of combination and invention, from the exchange of opinions, from the self-discipline which comes from sitting on councils and committees, from the courteous give-and-take of debate, from the self-reliance fostered by the management of corporate finances. It is not very easy to develop these qualities by attendance at meetings of a central society. Nurses who have already practised the art of governing a local centre are likely to take far more interest in the internal affairs of their College than those who attend merely as spectators in the hope of hearing a telling speech, but are terrified at a request to second a resolution or propose a vote of thanks. The local centre is a necessary training-ground for those who will one day perhaps be called to sit on the Central Council and committees. It will not do to leave London alone among the big towns, unprovided with this admirable educational stimulus in the art of organisation.

Let it not be thought that the establishment of London centres would be likely in any way to sap the influence and authority of the central management. Such centres would in effect prepare so many receptacles into which the College could pour at will the benefits desired by the entire profession. Managed on broad lines as shall hereafter be found most productive of good to all concerned, such offshoots would radiate back to the centre the light and heat they themselves receive.

